# Multi-omics analysis of expression and prognostic value of NSUN members in prostate cancer

**DOI:** 10.3389/fonc.2022.965571

**Published:** 2022-08-01

**Authors:** Guangyu Sun, Shenfei Ma, Zhiwen Zheng, Xiaohua Wang, Shuaiqi Chen, Taihao Chang, Zhengxin Liang, Yuchen Jiang, Shengxian Xu, Ranlu Liu

**Affiliations:** Department of Urology, Tianjin Institute of Urology, The Second Hospital of Tianjin Medical University, Tianjin, China

**Keywords:** prostate cancer, immune cell infiltration, mutation, NSun2, 5-Methylcytosine (5mC)

## Abstract

**Background:**

Prostate cancer is the most common tumor in men worldwide, seriously threatening the health of older men, and 5-methylcytosine (m5C) RNA modification has been shown to have a significant impact on the development and progression of various tumors. However, as the most critical methyltransferase for m5c RNA modification, the role of the NSUN members (NSUN1-7) in prostate cancer is unclear.

**Methods:**

We obtained sequencing data of genes and related clinical data of prostate cancer from The Cancer Genome Atlas (TCGA) database and Gene Expression Omnibus (GEO) database and analyzed the correlation between NSUN members’ expression and prognosis. we found that NSUN2 was closely implicated in the prognosis of prostate cancer, then verified the expression of NSUN2 in clinical samples, and obtained the correlation between NSUN2 and immune cell infiltration through CIBERSORT algorithm and ESTIMATE method. The relationship between NSUN2 copy number variation and immune cell infiltration was further analyzed in the TIMER database and identified signaling pathways associated with NSUN2 expression by GO, KEGG, and GSEA analysis. Finally, we verified the expression of NSUN2 in prostate cancer cell lines and confirmed the role of NSUN2 on the biological behavior of prostate cancer cells by proliferation and migration-related assays.

**Results:**

NOP2 and NSUN2 were upregulated in prostate tumor tissues. NSUN2 expression is closely associated with tumor prognosis. NSUN2 high expression implies poor clinical features, and the NSUN family is significantly associated with tumor stromal score and immune score. Besides, NSUN2 is associated with a variety of immune infiltrating cells (B cells memory, T cells CD4 memory resting, T cells CD4 memory activated, NK cells resting, and so on). High NSUN2 expression lowers the sensitivity of many chemotherapy drugs, such as docetaxel, doxorubicin, fluorouracil, cisplatin, and etoposide. In prostate cancer, the most common type of mutation in NSUN2 is amplification, and NSUN2 copy number variation is closely associated with NSUN2 expression and immune cell infiltration. GSEA analysis showed that the related genes were mainly enriched in ubiquitin-mediated protein hydrolysis, cell cycle, RNA degradation, endometrial cancer, prostate cancer, p53 signaling pathway, and NSUN2 potentiated the proliferation and migration of prostate cancer cells.

**Conclusions:**

NSUN2 is highly expressed in prostate cancer, which contributes to the progression of prostate cancer, and is closely implicated in immune cell infiltration and chemotherapy drugs. NSUN2 is expected to be a prospective marker and a new treatment target for prostate cancer.

## Introduction

5-methylcytosine (m5C) RNA modification has been a key post-transcriptional modulator of gene expression process recently, which is widely present in cells and has important roles in various biological metabolic processes (1). M5C gene has been described to be implicated in various human pathological processes such as neurological disorders and metabolism-related diseases (2–4). In addition, it has also been demonstrated that m5C gene abnormalities cause tumorigeneses, such as breast cancer, gallbladder cancer, and bladder cancer (5–7). However, as the most critical methyltransferase for m5c RNA modification, the role of the NSUN members (NSUN1-7) in prostate cancer is obscure.

We analyzed the expression of the NSUN members in tumor tissues, explored their relationship with patient prognosis and clinicopathological features, further analyzed the correlation between NSUN2 and immune infiltration of prostate tissues, and demonstrated that NSUN2 has an important effect on the proliferation and invasion of prostate cancer cells, which is related to the sensitivity of chemotherapy drugs, and NSUN2 is promising as a novel marker for predicting patient prognosis and therapeutic target.

## Methods

### Datasets source

The RNA-seq transcriptome data and clinical information on prostate cancer were downloaded from The Cancer Genome Atlas (TCGA), excluding patients with incomplete clinical information (8). There were 389 PRAD cases and 51 normal adjacent tissues in our study and for external validation, we downloaded 50 RNA-seq transcriptome data from Gene Expression Omnibus (GEO, GSE46602) (9).

Drug sensitivity data was obtained from The Genomics of Drug Sensitivity in Cancer (GDSC), collated using the R package “pRRophetic”.

### Differential expression analysis and correlation analysis of NSUN members

Differential expression of the NSUN members between PRAD and normal tissues was screened using the Wilcoxon test method. The heatmap and vioplot were performed respectively by the “heatmap” package and the “vioplot” package visualizing the expression levels and differences, with p < 0.05 considered statistically different.

### Analysis of the correlation between NSUN members and patients’ overall survival (OS), progression-free survival (PFS), and disease-free survival (DFS)

We identified genes significantly associated with patients’ OS, PFS, and DFS by cox regression analyses, and then further validated the correlation of NOP2 and NSUN2 with patients’ prognosis through Kaplan Meier analysis (10, 11).

### Protein-protein interaction network and immune-related scores of NSUN members

To better understand the interaction between the NSUN members we mapped the interaction network of 7 proteins through the STRING database (12), then calculated the relationship between NSUN1-7 and immune-related scores by ESTIMATE (13).

### Correlation analysis of NSUN2 and prostate cancer clinical features and immune infiltration

We focused on the association of NSUN2, a core gene in the NSUN members, with clinical features of prostate cancer, including tumor stage, Gleason score, and biochemical recurrence, and explored its relationship with immune cell infiltration by SSGSEA, CIBERSORT (14, 15).

### Mutation analysis of NSUN2

We obtained NSUN2 mutations in four datasets through the cBioPortal database (16), analyzed NSUN2 mutations with NSUN2 mRNA expression, and also examined the association between NSUN2 copy number variants and immune infiltrating cells in the TIMER database (17).

### GO, KEGG, GSEA functional enrichment analysis

Firstly, we found NSUN2-related differential genes by R package DESeq2, and the selection criteria were p < 0.05 and |log_2_foldchange| > 1. Thus, we conducted GO, KEGG, and GSEA functional analysis of differential genes separately though the R package “ClusterProfiler” (18).

### Clinical specimens and cell lines

Prostate cancer and the corresponding paired adjacent tissues were obtained from surgical specimens from the Department of Urology, Second Hospital of Tianjin Medical University.

Human prostate cancer cell lines LNCaP, C4-2B, PC-3, DU-145, and normal prostate cell line RWPE-1 were frozen, resuscitated, and cultured by Tianjin Institute of Urology.

### Cell siRNA transfection

Prepare the transfection reagent: A liquid: 500ul of serum-free medium (opti-MEM) + 10ul of Roche siRNA transfection reagent, B liquid: 500ul of serum-free medium (opti-MEM) + 10ul of siRNA, let the prepared A liquid stand at room temperature for 5min and then mix it with B liquid to obtain C liquid, let C liquid stand at room temperature for 20min.

Remove the culture dish from the cell incubator, add 1000ul of the above C solution to each well, and gently mix the C solution with 1640 in the well.

Put the culture dish back into the cell incubator and continue to incubate for 6h, then change the medium. The small interfering RNA (si-NSUN2-F,5’- UUCUCCGAACGUGUCACGUTT-3; si-NSUN2-R,5’ACGUGACACGUUCGGAGAATT-3’) was produced by GenePharma company.

### Extracted RNA, reverse transcribed, and real-time quantitative PCR (qRT-PCR)

Total RNA was extracted with Trizol reagent and SuperScript III RT Reverse Transcription Kit (ABI-Invitrogen) was used for cDNA synthesis. The qRT-PCR reaction was performed by SYBR qPCR Mix (ABI-Invitrogen).

Primer sequences: NSUN2-F,5′-GAACTTGCCTGGCACACAAAT-3′; NSUN2-R, 5′- TGCTAACAGCTTCTTGACGACTA-3′;

### Immunohistochemistry

Our study was approved by the Ethics Committee of the Second Hospital of Tianjin Medical University. Written informed consent was obtained from all participants. Our team collected 16 prostate tumor tissue samples and 16 benign prostate tissue samples, sliced them up into 4mm sections, and stained them with Zhongshan Jinqiao immunohistochemistry kit pv6001.

### Cell counting Kit-8 (CCK-8) assay

Evaluation of prostate cancer cell proliferation through the CCK-8 kit.

2×10^3^ cells per well were inoculated into 96-well plates and incubated for 1, 2, 3, 4, and 5 days. Subsequently, add CCK-8 reagent and continue to incubate for 2h, the absorbance of each well was analyzed at 450 nm through an enzyme-linked immunoassay analyzer.

### 5-ethynyl-2’-deoxyuridine (EDU) proliferation assay

Prostate cancer cell proliferation was assessed through the Abbkine EDU kit.

2×10^4^ cells per well were inoculated into 24-well plates and incubated for 24 h. Subsequently, EDU working solution was added for labeling and incubation continued for 2h. Cells were then fixed and permeabilized with formaldehyde and triton reagent, followed by incubation with 500ul Click-iT mixture for 30 minutes, then DAPI was added for nuclear staining, and finally photographed and counted by electron fluorescence microscopy.

### Scratching assay

Inoculate cells into 6-well plates, the cell confluence was 80-100%, the bottom of the six-well plates was scribed vertically with a 10ul gun tip, and the gun tip was changed every time to avoid the uneven width of each scratch when the gun tip became blunt. Rinse the cells gently with PBS 3 times to remove the non-adherent cells after scribing, and replace the basal medium without serum. The cells were incubated in a 37°C, 5% CO2 incubator. Images of the cell trabeculae were taken by microscope at 0h, 24h, and 48h after scribing, respectively.

## Results

### The expression landscape of NSUN members in PRAD

**Figure 1:**
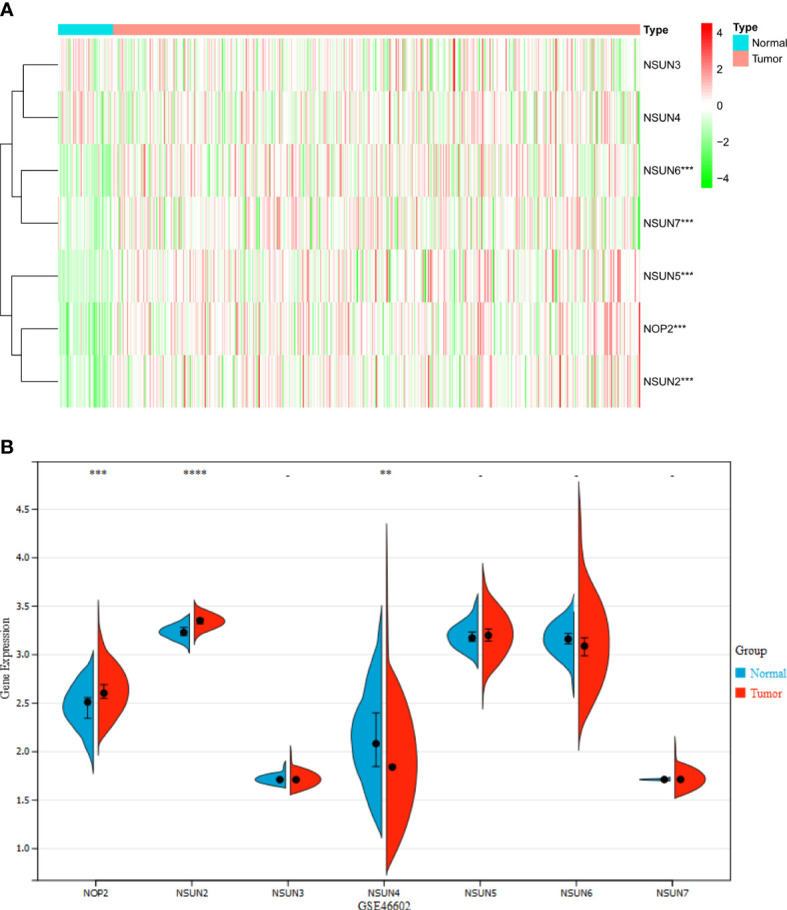
The Expression landscape of NSUN members in PRAD (**P <0.01; ***P<0.001; ****P<0.0001). **(A)** Heatmap of the expression of NSUN members in the TCGA dataset. **(B)** Violin plot of the expression of NSUN members in the GSE46602 dataset.

There were seven genes included in the landscape of NSUN members in PRAD. Heatmap clearly revealed these genes were differentially expressed between 389 PRAD and 51 normal prostate samples through the TCGA database (**Figure 1A**). NOP2, NSUN2, NSUN5, NSUN6, and NSUN7 were highly expressed in PRAD tissues. In addition, we also analyzed the expression of NSUN members in GSE46602, NOP2 and NSUN2 were highly expressed in tumor tissues, and NSUN4 was lowly expressed (**Figure 1B**).

### Relationship between NSUN members and OS, DFS, PFS

**Figure 2:**
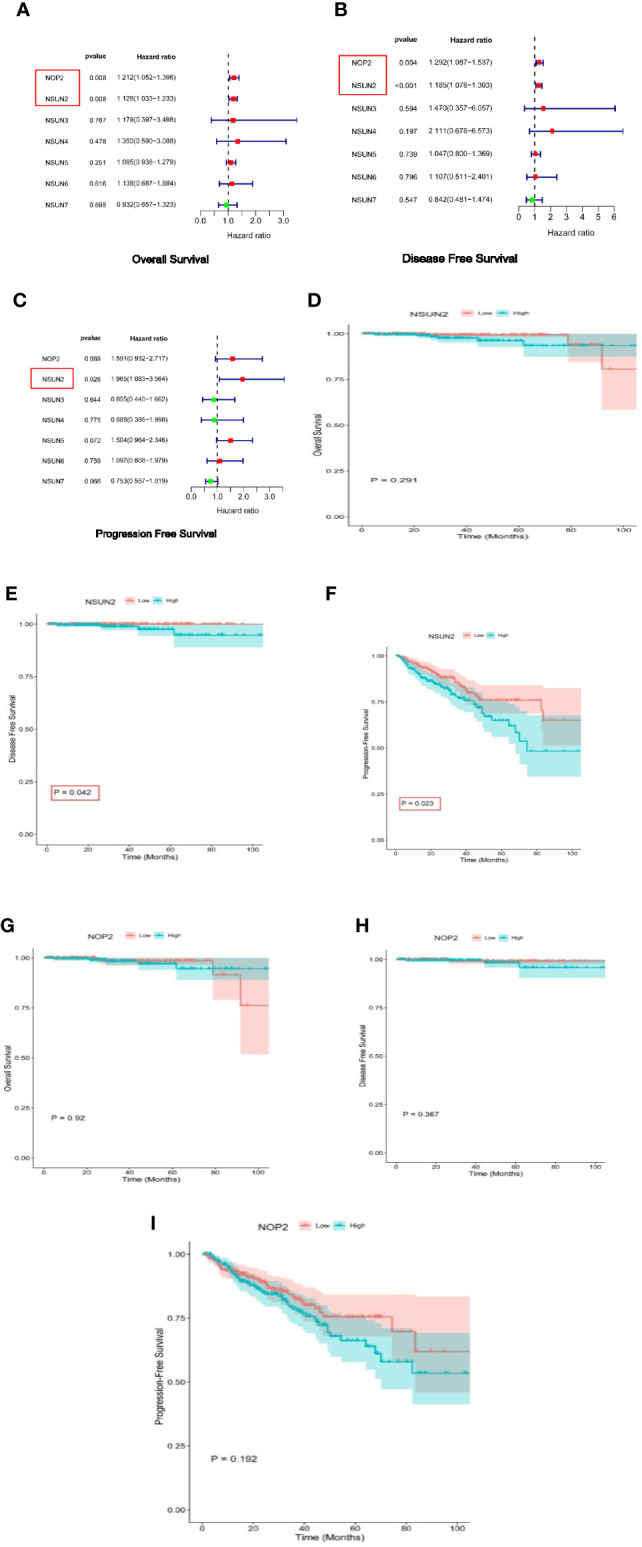
Survival analysis of NSUN members in PRAD. **(A–C)** Univariate Cox regression analyses of NSUN members in PRAD(overall survival, disease-free survival, and progress-free interval survival). **(D–F)** Kaplan-Meier survival curves comparing the high and low expression of NSUN2 in PRAD. (overall survival, disease-free survival, and progress-free interval survival). **(G–I)** Kaplan-Meier survival curves comparing the high and low expression of NOP2 in PRAD. (overall survival, disease-free survival, and progress-free interval survival).

We investigated the relationship between the NSUN members and the OS rates, as well as DFS and PFS. As a result, NOP2 and NSUN2 were significantly associated with OS and DFS (**Figures 2A, B**). Only NSUN2 was associated with PFS **Figure 2C**). We then verified through the KM method and found that NSUN2 was significantly associated with PFS, and DFS in patients (**Figures 2D–I**).

### PPI network and immune-related scores of NSUN members

**Figure 3:**
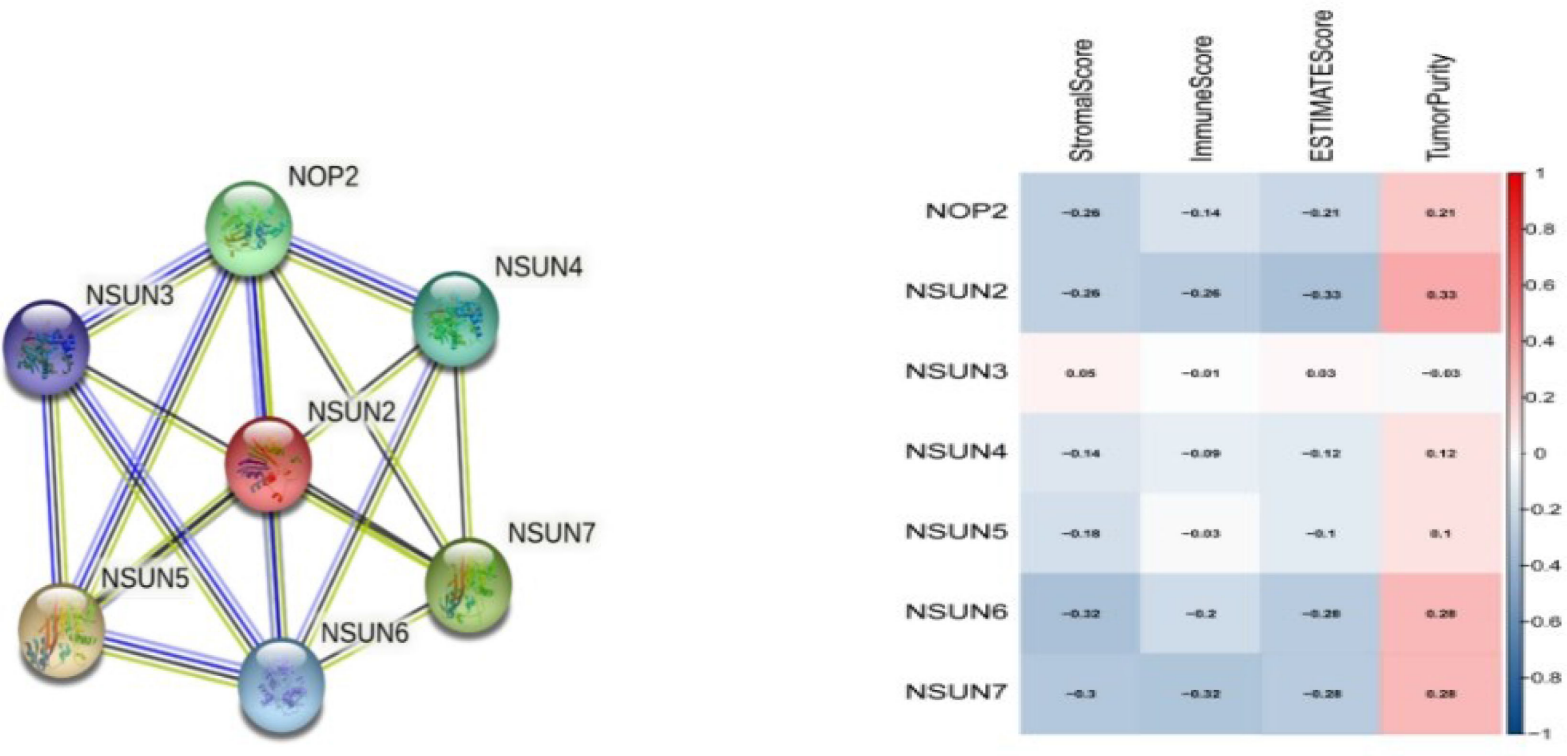
protein-protein interaction (PPI)network and Immune-related scores of NSUN members. **(A)** A network diagram of interactions between proteins encoded by genes of the NSUN members(PPI enrichment p-value < 1.0e-16). **(B)** The correlation analysis among NOP2/Sun family members with Immune-related scores.

We used the STRING database to map the protein-protein interacted (PPI) network of NSUN members and found that they were closely interconnected (PPI enrichment p-value < 0.0001), with NSUN2 at the core of the network (**Figure 3A**). Then we analyzed the immune-related score for each prostate cancer case by ESTIMATE and found that NSUN2 had the highest correlation coefficient with the immune score and tumor purity (**Figure 3B**), so we suggested that NSUN2 might be a key gene in prostate cancer and explored it further in-depth subsequently.

### Correlation of NSUN2 expression and clinical characteristics

**Figure 4:**
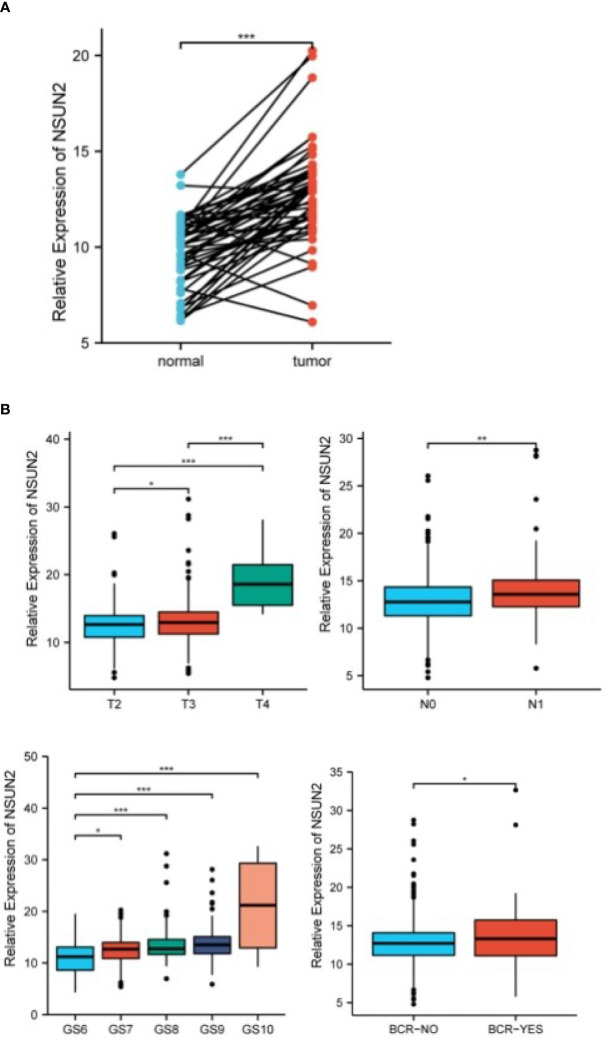
NSUN2 in prostate tumor tissue and normal tissue, and its correlation with clinical features (*P<0.05; P **<0.01; ***P<0.001). **(A)** NSUN2 expression in prostate tumors and paired normal tissues (TCGA database, 51 pairs). **(B)** Expression of NSUN2 in different tumor stages, lymph node metastasis, Gleason score, and biochemical recurrence or not.

We compared the expression of NSUN2 in 51 pairs of tumor and adjacent paired samples through the TCGA database, and found that NSUN2 was upregulated in tumors and associated with higher tumor stage and biochemical recurrence risk (**Figures 4A–C**).

### Immune infiltration analysis

**Figure 5:**
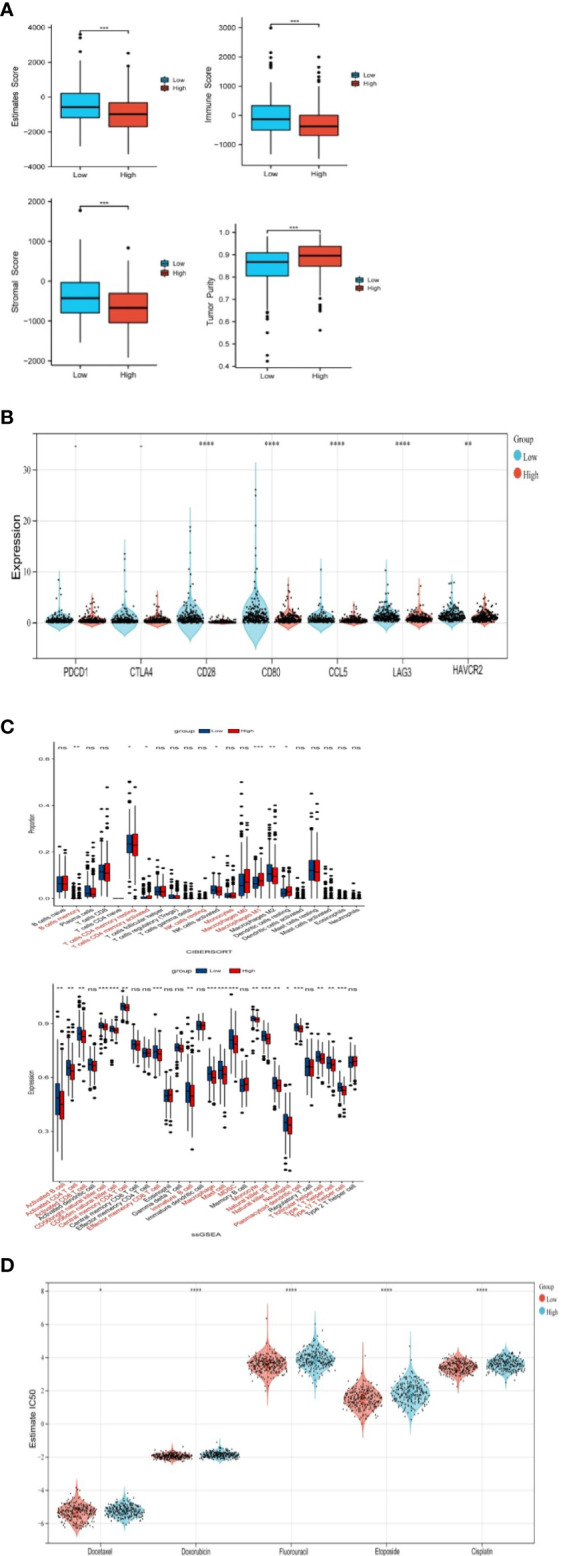
Correlation of NSUN2 with immune cell infiltration(*P<0.05; P **<0.01; ***P<0.001; **** P<0.0001; ns, no significance). **(A)** Differences in immune-related scores between two groups with high and low expression of NSUN2 through ESTIMATE. **(B)** Association of NSUN2 with immune checkpoint genes. **(C)** Differences in immune cell infiltration in NSUN2 high and low expression groups through CIBERSORT and SSGSEA. **(D)** Effect of NSUN2 on the IC50 of oncological chemotherapy drugs.

Estimate analysis showed that StromalScore, ImmuneScore, and EstimateScore of the NSUN2 high expression group were lower than that of the low expression group, and the TumorPurity was higher than that of the low expression group (**Figure 5A**).

In addition, we also analyzed different immune checkpoint-related genes in NSUN2 high and low expression groups, we found that the differences in the expression of conventional immune checkpoint genes PDCD1, and CTLA4 were not significant in the two groups, but the expression of novel immune checkpoint genes (CD28, CD80, CCL8, LAG 3, HAVCR2) was significantly higher in NSUN2 low expression groups (**Figure 5B**).

CIBERSORT showed the proportion of 22 kinds of immune cell infiltration in the low expression group and high expression group (**Figure 5C**). B cells memory, T cells CD4 memory resting, T cells CD4 memory activated, NK cells resting, Monocytes, Macrophages M0, and Macrophages M2 were significantly different in the two groups.

SSGSEA likewise compared the differences in immune cell infiltration between the two groups, with a higher proportion of many immune cells in the NSUN2 low expression group, such as activated B cell, activated CD4 T cell, and activated CD8 T cell (**Figure 5D**).

In addition, we analyzed the effect of NSUN2 on the IC50 of chemotherapy drugs for prostate cancer by using gene-drug response data from the GDSC database and found that NSUN2 had a significant effect on chemotherapy drugs commonly used in the clinical setting, including docetaxel, doxorubicin, fluorouracil, cisplatin, and etoposide. In the group with high NSUN2 expression, patients had low sensitivity to chemotherapy. Therefore, we suggest that NSUN2 may contribute to the immune cells in TME and is nearly associated with chemotherapeutic drug resistance.

### Mutation analysis of NSUN2

**Figure 6:**
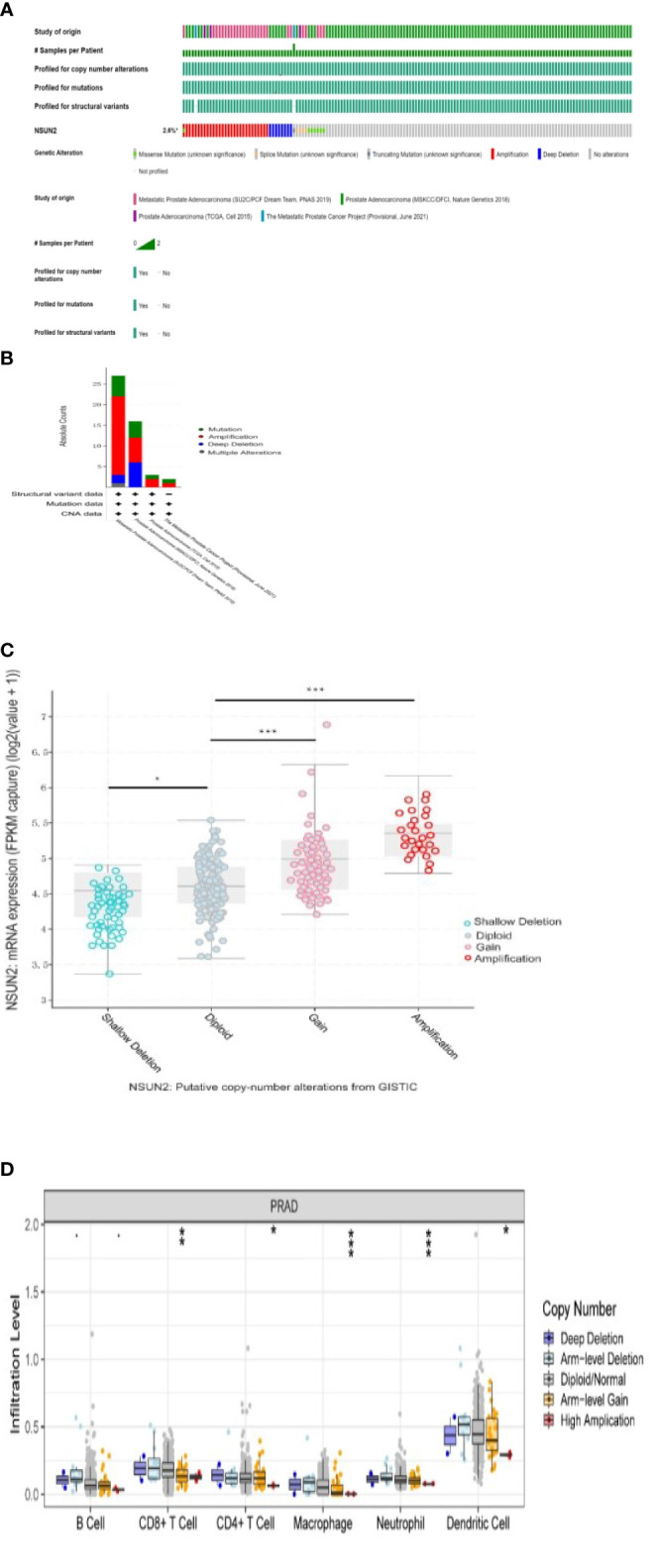
Analysis of NSUN2 mutations in prostate cancer(*P<0.05; P **<0.01; ***P<0.001). **(A, B)** NSUN2 mutations in 1873 prostate patients from the cBioPortal database. **(C)** Effect of NSUN2 copy number variation on gene expression through cBioPortal database. **(D)** Effect of NSUN2 copy number variation on immune cell infiltration by TIMER database.

We analyzed NSUN2 mutations in four datasets with 1873 patients. The mutation frequency of NSUN2 reached 2.6%, the most common mutation type was amplification (**Figures 6A, B**), NSUN2 copy number variation was closely associated with NSUN2 expression, and both NSUN2 amplification and gain can lead to elevated NSUN2 expression (p<0.05, **Figure 6C**).

In addition, we evaluated the effect of NSUN2 mutations with different copy numbers on immune infiltration by the TIMER database and found differences in CD8+ T cell, CD4+ T cell, macrophage, neutrophil, and dendritic cell (**Figure 6D**).

### GO, KEGG, GSEA functional enrichment analysis

**Figure 7:**
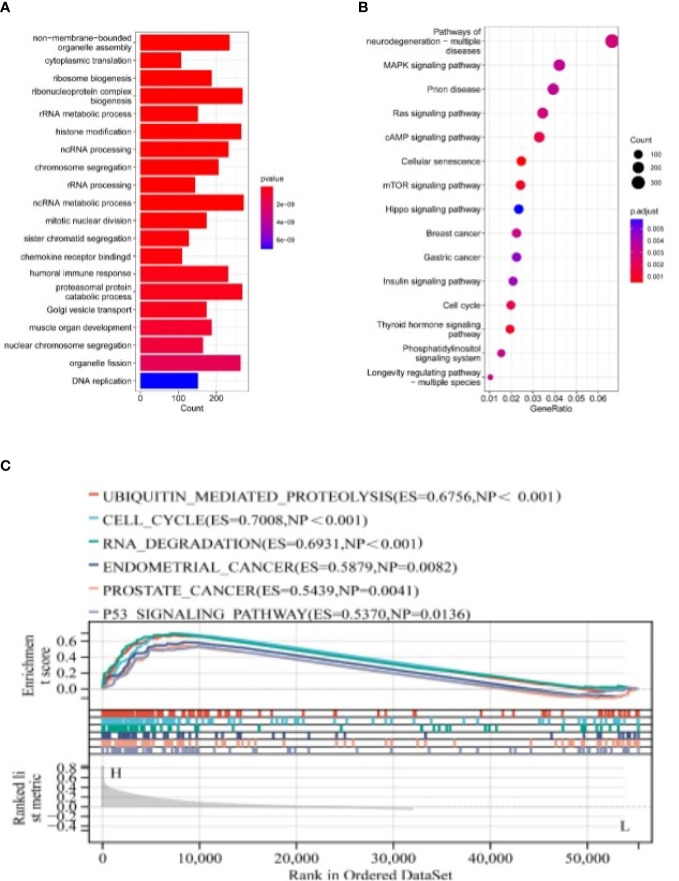
The Pathways Regulated by NSUN2 through GO, KEGG, and GSEA Analyses. **(A, B)** GO and KEGG analyses of NSUN2. **(C)** GSEA analysis of NSUN2.

GO analysis suggested that differential genes are enriched in a wide range of organisms’ metabolic activities, such as non-membrane-bounded organelle assembly, histone modification, humoral immune response, and chemokine receptor binding (**Figure 7A**).

KEGG analysis suggested that differential genes were significantly enriched in Pathways of neurodegeneration - multiple diseases, MAPK signaling pathway, mTOR signaling pathway, Hippo signaling pathway, and other signaling pathways (**Figure 7B**).

We then performed GSEA analysis on the differential genes and found that the genes were related to multiple disease pathways, such as ubiquitin-mediated protein hydrolysis (ES=0.6756, NP<0.001), cell cycle (ES=0.7008, NP<0.001), RNA degradation (ES=0.6931, NP<0.001), endometrial cancer (ES=0.5879, NP=0.0082), prostate cancer (ES=0.5439, NP=0.0041) and p53 signaling pathway (ES=0.5370, NP=0.0136) (**Figure 7C**).

### Validation of NSUN2 expression and function in prostate cancer

**Figure 8:**
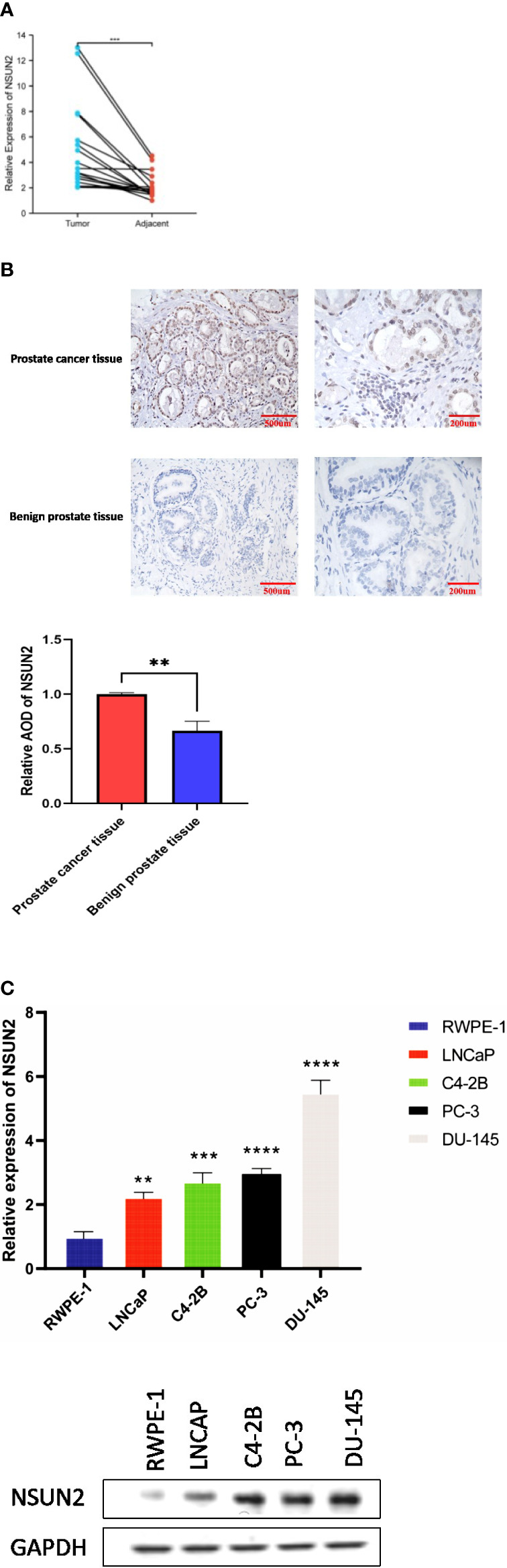
NSUN2 expression in prostate cancer clinical tissues and cell lines (P **<0.01; ***P<0.001; **** P<0.0001). **(A)** Expression of NSUN2 in 16 pairs of prostate cancer and adjacent tissues through qPCR. **(B)** Expression of NSUN2 in benign prostate tissues and prostate cancer tissues through Immunohistochemistry. **(C)** Expression of NSUN2 in normal prostate epithelial cell line and different prostate cancer cell lines through qPCR and western blot.

We determined the expression of NSUN2 through qPCR and western blot in a prostate normal epithelial cell line and four prostate cancer cell lines. In addition, we analyzed the expression of NSUN2 in prostate cancer tissues and normal tissues by qPCR and immunohistochemistry, the expression of NSUN2 was elevated in prostate cancer. (**Figures 8A–C**).

**Figure 9:**
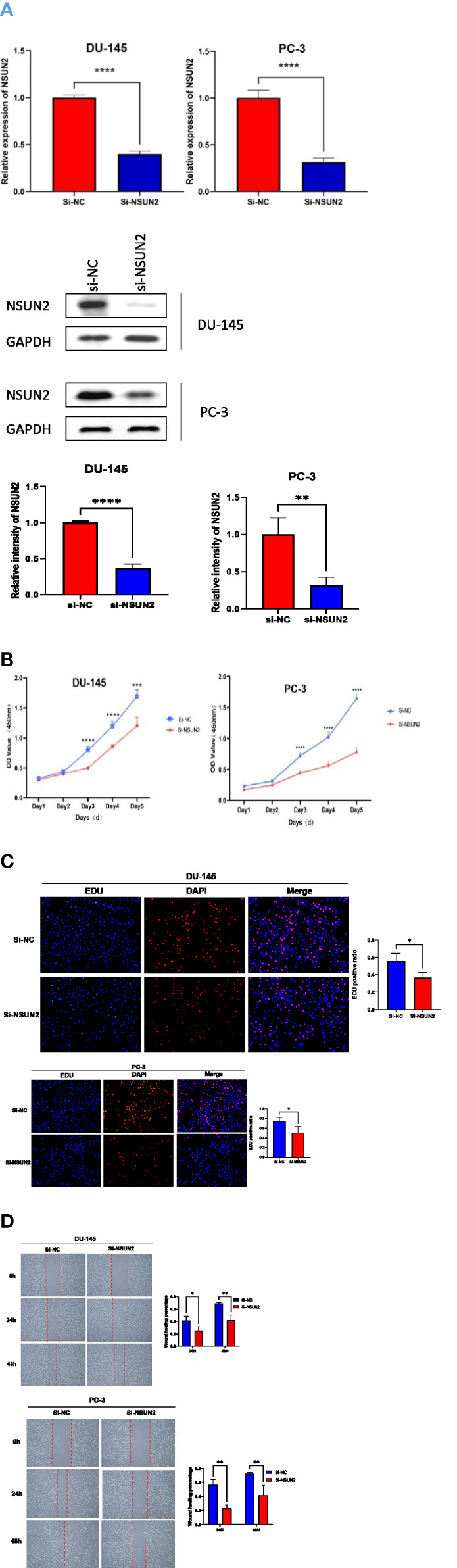
NSUN2 promotes the proliferation and invasion of prostate cancer cells(*P<0.05; P **<0.01; ***P<0.001; **** P<0.0001). **(A)** QPCR and western blot showed the efficiency of knocking down NSUN2 in PC-3 and DU-145 cells. **(B)** CCK8 proliferation assay of DU-145 and PC-3 cells. **(C)** EDU proliferation assay of DU-145 and PC-3 cells. **(D)** Scratching assay of DU-145 and PC-3 cells.

We knocked down NSUN2 by siRNA in the PC-3 cell line and DU-145 cell line (**Figure 9A**). CCK8 assay, EDU assay, and scratching assay showed that NSUN2 contributed to the proliferation and migration of tumor cells (**Figures 9B–D**).

## Discussion

With the rapid development of the world economy, the increasing aging of the population, and the popularity of PSA screening, the incidence of prostate cancer is rapidly increasing. According to the International Cancer Center, prostate cancer has the highest incidence and the second-highest mortality rate among male cancers, second only to lung cancer (19). However, due to the insidious nature of early symptoms of prostate cancer and differences in prostate-specific antigen (PSA) screening in different regions, approximately 70% of patients in China are initially diagnosed with high-risk progressive prostate cancer or even advanced prostate cancer (20, 21). Therefore, it is required and urgent to explore new useful markers for diagnosis and prognosis. Professor Alanne identified PIAS3 as a potential biomarker for Gleason score 4 + 3 = 7 for prostate cancer and UBE2V2 as a potential biomarker for Gleason score 6, while Professor ElKarami created a model for predicting upgrading on magnetic resonance imaging targeted biopsy by machine learning (22, 23).

RNA m5c has a significant effect on life processes such as RNA translation, stabilization, nucleation, and DNA damage repair (24). Lately, an increasing number of studies have observed that tumorigenesis and development are influenced by m5C modifications (25). However, the function of m5C-related genes in PRAD is unclear.

Our work was the first to methodically analyze the expression of NSUN members in PRAD. With the TCGA dataset, we showed that the expression of NOP2, NSUN2, NSUN5, NSUN6, and NSUN7 were significantly increased in PRAD. In the GSE46602 dataset, we also found that NOP2 and NSUN2 were significantly increased, but NSUN4 was decreased in PRAD. Subsequently, we performed survival analysis, PPI interaction network analysis, and immune score analysis on the NSUN family. We found that NSUN2 was closely related to PFS, and DFS of patients, and was at the core of the PPI network with high immune-related scores, so we further analyzed NSUN2 in depth.

NSUN2 is an important nucleolar protein that has a great effect on tissue homeostasis and material transport and is also able to regulate cell proliferation by regulating cell cycle factors (26). In addition, NSUN2 has been found to promote the development and progression of bladder cancer (27). Another research showed that NSUN2 promoted the occurrence and development of hepatocellular carcinoma through m5C modification (28).

Our study revealed for the first time that NSUN2 is highly expressed in prostate cancer and is strongly associated with tumor stage, lymph node invasion, Gleason score, and biochemical recurrence. Furthermore, we also demonstrated that NSUN2 promotes the proliferation and migration of prostate cancer cells. To elucidate the functional roles associated with NSUN2, through functional enrichment analysis, we noticed those differential genes were enriched in a variety of metabolic activities, including cytoplasmic translation, mitosis, and histone modification, which is consistent with previous findings (29). More importantly, we found significant differences in several important tumors signaling pathways, mTOR signaling pathway, HIPPO signaling pathway, MAPK signaling pathway, and Ras signaling pathway, which suggests that NSUN2 does have an impact on the development and progression of prostate cancer.

The immune microenvironment is composed of many kinds of immune cells and biomarkers, it has been found that immune cell infiltration correlates closely with tumor stage and lymph node metastasis (30) and that high levels of immune cell infiltration usually mean that patients have a higher prognosis and a relatively high success rate of response to immunotherapy (31). A recent study found that NSUN2 promoted the progression of nasopharyngeal carcinoma by regulating immune infiltration (32), In our study, we also found that NSUN2 was closely associated with immune cell infiltration such as activated B cell, activated CD4 T cell, activated CD8 T cell, and NSUN2 expression also had a significant effect on immune checkpoint genes(CD28, CD80, CCL8, LAG3, HAVCR2), suggesting that NSUN2 may have an effect on the immune system through epigenetic modifications which could be a valid predictor of immunotherapy, but this needs to be further confirmed.

Chemotherapy is a major part of the treatment for prostate cancer. In clinical practice, docetaxel is the first-line treatment option for castration-resistant prostate cancer (33). However, chemoresistance usually occurs in the treatment of advanced tumors. We evaluated the correlation between NSUN2 expression and chemotherapy sensitivity and observed that patients with lower NSUN2 expression responded more sensitively to most chemotherapeutic agents, such as docetaxel and cisplatin, etc. This suggests that NSUN2 may provide new options for choosing appropriate chemotherapy drugs for prostate cancer patients.

Gene mutations lead to abnormal and uncontrolled cell growth, which is an important cause of tumor development and progression, and identifying gene mutations is critical to the precise treatment of tumors (34). We analyzed the mutation of NSUN2 in 1873 patients by cBioPortal database, the mutation frequency was 2.6%, the most common mutation type was amplification, and the alteration of NSUN2 copy number also had a role on gene expression and immune cell infiltration.

In conclusion, we showed the gene expression profile of NSUN members and identify NSUN2, a core gene for m5C modification, as a potential biomarker for predicting prostate cancer prognosis. Our work suggested that there may be a link between RNA m5C modification and prostate cancer immune cell infiltration, however, this needs to be further confirmed.

## Data availability statement

The original contributions presented in the study are included in the article/supplementary material. Further inquiries can be directed to the corresponding author.

## Author contributions

All authors listed have made a substantial, direct, and intellectual contribution to the work, and approved it for publication.

## Funding

This research was supportede by the Tianjin ad technology plan project (19ZXDBSY00050).

## Conflict of interest

The authors declare that the research was conducted in the absence of any commercial or financial relationships that could be construed as a potential conflict of interest.

## Publisher’s note

All claims expressed in this article are solely those of the authors and do not necessarily represent those of their affiliated organizations, or those of the publisher, the editors and the reviewers. Any product that may be evaluated in this article, or claim that may be made by its manufacturer, is not guaranteed or endorsed by the publisher.
